# Supplementary material for: A model of tension-induced fiber growth predicts white matter organization during brain folding
Source: Nat Commun. 2021 Nov 18;12:6681. doi: 10.1038/s41467-021-26971-9 (PMC8602459; doi:10.1038/s41467-021-26971-9)
Supplement: Supplementary file 1 — Supplementary Information [file 41467_2021_26971_MOESM1_ESM.pdf]

A model of tension-induced fiber growth predicts white matter organization during brain folding

Kara E. Garcia, Xiaojie Wang, Christopher D. Kroenke

Supplementary Note 1. Effect of subplate heterogeneities on cortical folding

For the folding simulations described in Figs. 2-4, symmetry breaking to induce the first fold was accomplished by introducing a small region of slightly increased cortical growth. However, a wide range of geometric and subplate perturbations would also be capable of breaking symmetry to induce the first fold in our simulations, as described in Supplementary Fig. 1.

Supplementary Fig. 1a corresponds to the ellipsoidal case from the main text (matching Fig. 6a, aspect ratio = 1.1), extended to later time points. While no folding is observed prior to T=1, the curvature variation present in ellipsoidal models is sufficient to induce folding at slightly later time points. Similar to the folding cases described in the main text, folding under this perturbation induces the same stereotyped subplate organization relative to folds, with predominantly radial fibers beneath gyri and predominantly tangential fibers beneath sulci (Supplementary Fig. 1a).

For the same model, we also considered the effect of a local symmetry-breaking perturbation in the subplate, in which the initial fiber orientation has been altered in a very small region, shifting from the weakly radial orientation ($f_{1,0}$=0.12 and $f_{2,0}$=$f_{3,0}$=0.1) to a weakly tangential orientation ($f_{1,0}$=0.105, $f_{2,0}$=0.115, $f_{3,0}$=0.1). The total initial fiber volume fraction remains unchanged throughout the subplate in this example. As shown in Supplementary Fig. 1b, this perturbation is sufficient to induce a sulcus in the location that would have otherwise produced a gyrus. Since stress-induced fiber reorganization is expected to encourage tangential fiber growth beneath sulci, the initial fiber orientation in Supplementary Fig. 1b complements the folding-induced patterns of subplate organization. However, introduction of a third, competing bias – locally increased cortical growth to induce a gyrus, as described for simulations in the main text – is able to override the bias from fiber orientation and induce a gyrus. In this case (Supplementary Fig. 1c), stress-induced radial fiber growth beneath the gyrus overrides the original tangential subplate orientation. The resulting final subplate orientation is consistent with stereotyped trends relative to gyri and sulci described throughout this study.

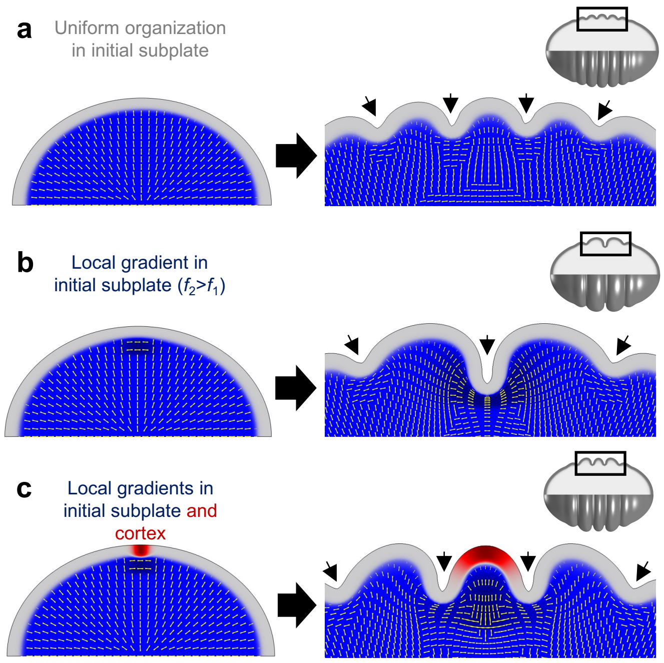

Supplementary Figure 1. Subplate organization with alternative symmetry-breaking perturbations to induce folding. For various perturbations (a-c), initialized model (T=0.0) is shown to left and final result (T=1.25) is shown to right. In all images, cortex is shown in gray (or red) and subplate is shown in blue, with yellow tick marks indicating the primary direction of fiber orientation. In panels b-c, dark blue region indicates location and extent of the local shift in initial subplate organization. In panel c, red region indications the location and extent of the local increase in cortical expansion. In all cases, final subplate fiber organization is consistent with trends predicted for the spherical cases in Fig. 2-4, with radial organization beneath gyri and tangential organization beneath sulci. **(a)** Curvature variation in an ellipsoid (initial aspect ratio = 1.1 shown here) provides sufficient perturbation to allow buckling, with gyrus in center. **(b)** Addition of nonuniformity in the initial subplate orientation is sufficient to bias formation of a sulcus instead of gyrus in center, with resulting fiber organization reinforcing the initial subplate pattern. **(c)** Further addition of local increase in cortical growth overrides bias from (b) to induce formation of a gyrus. Resulting fiber organization contradicts initial subplate pattern.

The influence of various symmetry-breaking fiber orientations was explored further in Supplementary Table 1. To understand the influence of fiber orientation in the absence of competing geometric or growth perturbations, simulations returned to an initial spherical geometry with no local increase in cortical expansion.

Under both low and high curvatures, a local predominance of radial fibers (direction 1, increased radial growth in response to tension) led to local formation of a gyrus, while a local predominance of circumferential fibers (direction 2, increased tangential growth in response to tension) led to formation of a sulcus. A local predominance of tangential fibers along the axis of symmetry (direction 3, increased meridional growth in response to tension) induced formation of a gyrus in high curvature models (initial geometry in Fig. 2a), but a sulcus in low curvature models. For the case of high curvature, this can be understood as increased growth in response to hoop stress, such that increased growth of a ring around the axis of symmetry will form a gyrus. However, this effect weakens with increasing radius, such that models of very low initial brain curvature exhibit a different trend (indicated with asterisk).

In cases where a localized increase in fibers of one direction came at the expense of fibers in the other directions, these trends were more pronounced. In the reverse scenarios, a local decrease in radial direction 1 or axisymmetric direction 3 fiber content led to formation of a sulcus, due to a localized decreased gyrus-inducing growth. By contrast, when only circumferential direction 2 (sulcus-inducing) fibers have been reduced, growth is biased to form a gyrus.

Supplementary Table 1. Predicted fold morphology above variation in subplate fiber distribution

Local fiber volume fraction relative to surrounding subplate	Initial predominant fiber orientation	High curvature model prediction	Low curvature model prediction
Higher or Same	1 > 2 = 3	Gyrus	Gyrus
	2 > 1 > 3	Sulcus	Sulcus
	3 > 1 > 2	Gyrus*,+	Sulcus*,+
Lower or Same	2 = 3 > 1	Sulcus+	Sulcus+
	1 > 3 > 2	Gyrus	Gyrus
	1 > 2 > 3	Sulcus*	Gyrus*

*predicted direction of folding depends on initial model curvature

+stress-induced remodeling deviates from the initially predominant fiber orientation

For the majority of scenarios, stress-induced fiber remodeling, as predicted to result from gyrus or sulcus formation, propagates the initial discrepancy in fiber orientation, suggesting a positive feedback effect. In these cases, models proposing folding as either the cause or effect of fiber organization could accurately predict the relationship between subplate organization and final morphology. However, Supplementary Table 1 highlights several instances, denoted by (+), where stress-induced remodeling is expected to deviate from the initially predominant fiber orientation and has the potential to override initial organization.

Supplementary Note 2. Effect of ECM production on folding and fiber organization

The constitutive growth model described in Methods allows fiber elongation to continue indefinitely, as long as tension is maintained above the target stress. Since non-fiber components of the subcortical tissue are set to be nongrowing, this theory allows fiber volume fraction to approach 100% as time approaches infinity. However, fiber volume fraction in the adult white matter is known to be closer to 80%, with extracellular matrix (ECM) accounting for approximately 20% of tissue volume. While models considered in the primary analysis of this study remain well under this threshold at final time points, here we consider an alternative model in which a small amount of ECM is generated alongside fiber elongation.

Supplementary Fig. 2 describes the impact of this additional term for both the theoretical case described in Fig. 8 and the primary model in Figs. 2-4. As shown in Supplementary Fig. 2a, addition of ECM lowers the maximum theoretical fiber volume fraction as time approaches infinity. In a full simulation of folding (Supplementary Fig. 2b), addition of ECM generation led to higher overall subplate growth in response to stress, requiring a significant decrease in the stress-dependent growth parameter, a. For the case shown, inclusion of 20% (0.2 unit volume) ECM generation for every unit volume of fibers generated required a 40% reduction in a to achieve folding at a similar time, T=1. The resulting increase

in isotropic growth produced flatter folds, as well as additional secondary folds along the perimeter of the model. The addition of isotropic tissue also decreased overall fractional anisotropy (FA). While fiber remodeling was slightly weaker due to the reduction in *a*, the same pattern was observed relative to gyri and sulci.

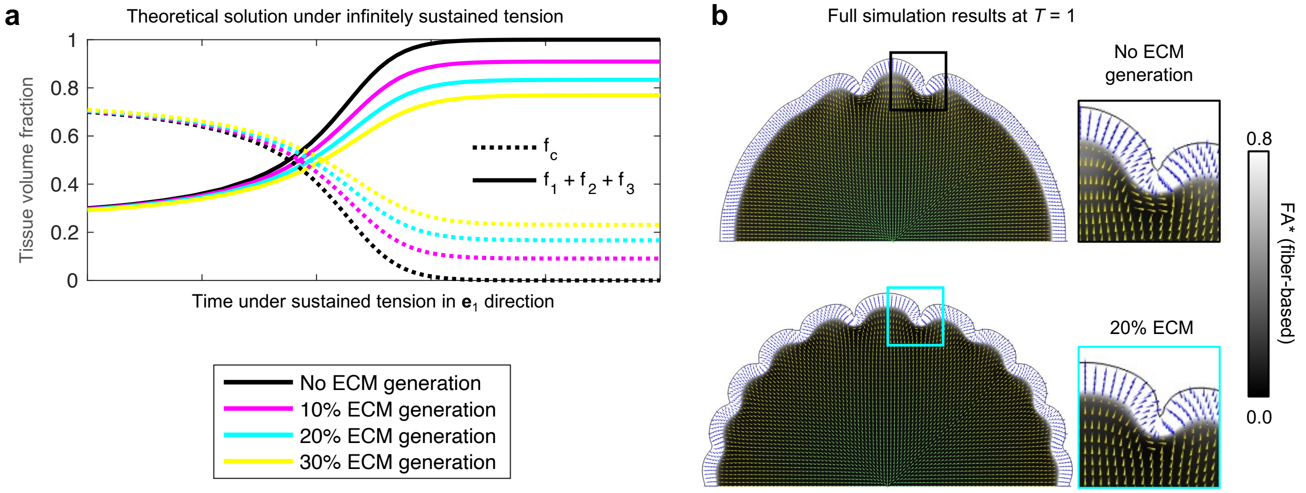

Supplementary Figure 2. Evolution of subcortical fibers under the condition of stress-dependent extracellular matrix generation. **(a)** In the theoretical case of sustained tension and fiber elongation (as time approaches infinity), we considered an additional ECM volume generation of 10%, 20%, or 30% for every unit volume of fibers generated. This approach leads to predictable maximum thresholds for fiber volume fraction as time approaches infinity (90%, 80%, and 70%, respectively). In the examples shown, initial fiber volume fraction in each direction was set to 10% (f_1=f_2=f_3=0.1), such that the initial volume fraction of non-fiber components (f_c) was 70%. **(b)** Full folding simulation with no ECM generation (top) and 20% ECM generation (bottom). FA* maps are based on the 3D tensor describing model subplate volume fractions, with cortical FA* set to 1 for visualization. Tick marks indicating the primary direction of fiber orientation are colored such that blue = cortex, yellow = subplate and deeper subcortical layers.

Supplementary Note 3. Effect of linear versus exponential cortical growth

In the main analysis of this study, we assumed growth of the cortex to be exponential as a function of time, with exponential growth rate calibrated from cortical expansion measures, as described in Methods. However, many models (Bayly et al. 2013, Holland et al. 2015, Tallinen et al. 2016) have considered linear growth in each tangential direction, corresponding to quadratic surface area expansion as a function of time. To test the influence of this discrepancy, we considered an alternative model with cortical growth defined according to the equation $G_2 = G_3 = 1 + ct$, where c represents the linear cortical growth rate.

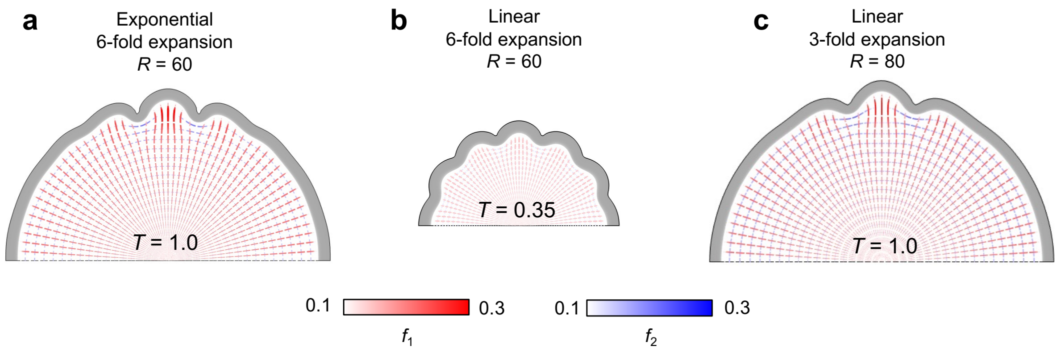

Supplementary Figure 3. Folding and subcortical fiber distributions under the condition of linear cortical growth. (a) Exponential cortical growth model with default *R*, from Figs. 2-4, for comparison. **(b)** For the same total cortical expansion (6-fold expansion in 50 days) and exponential fiber elongation rate, linear cortical growth leads to much earlier folding. **(c)** Under linear cortical growth, morphology and subcortical patterns similar to the exponential cortical growth model (a) can be achieved with lower total cortical expansion (3-fold expansion) and faster fiber elongation. For each case, simulation time (*T*) was chosen to illustrate morphology and fiber volume fractions at a similar stage of fold maturity. Crosses (+) are used to visualize fiber volume fraction in terms of radial (f_1) and tangential (f_2) directions. Fiber volume fraction is represented by color (radial fibers in red, tangential fibers in blue), such that a dark red region indicates increased radial fibers per unit volume, dark blue indicates increased tangential fibers per unit volume, and light color indicates relatively low fiber volume fraction.

Notably, stress-dependent subcortical growth defined here and in past studies (Bayly et al. 2013, Holland et al. 2015) is based on an exponential form. Thus, under a constant stress and exponential cortical growth, the nondimensionalized response ratio, *R,* directly relates two exponential growth rates: exponential fiber elongation in response to stretch divided by exponential cortex elongation in each tangential direction. Relative to an exponential growth curve, linear cortical growth exhibits much faster growth at early time points and slower growth at later time points. For a linear expansion defined to reach the same total expansion (approximately 6-fold increase in surface area over 50 days), we note that the original value for stress-dependent fiber elongation (*R*=60) produces much earlier folding (Supplemental Fig. 3b). Since the effective *R* is lower at the onset of folding in this case, the result can be understood in the same way as models of exponential cortical growth with very slow fiber elongation rates (Fig. 3b).

Supplemental Fig. 3c illustrates the case of reduced cortical expansion and faster fiber elongation, to achieve a similar degree of folding at *T*=1.0. In this way, cortical growth defined to occur linearly over time (but axon elongation occurs exponentially over time) facilitates folding under faster values of axon elongation. However, the primary effect of interest in this study – stereotyped organization of the subplate as the result of cortical folding stresses – is similar in both scenarios of linear cortical growth.
