# Supplementary material for: A model of tension-induced fiber growth predicts white matter organization during brain folding
Source: Nat Commun. 2021 Nov 18;12:6681. doi: 10.1038/s41467-021-26971-9 (PMC8602459; doi:10.1038/s41467-021-26971-9)
Supplement: Supplementary file 3 — Reporting Summary [file 41467_2021_26971_MOESM3_ESM.pdf]

nature research

Corresponding author(s): Kara Garcia

Last updated by author(s): Oct 15, 2021

Reporting Summary

Nature Research wishes to improve the reproducibility of the work that we publish. This form provides structure for consistency and transparency in reporting. For further information on Nature Research policies, see our Editorial Policies and the Editorial Policy Checklist.

Statistics

For all statistical analyses, confirm that the following items are present in the figure legend, table legend, main text, or Methods section.

n/a	Confirmed	
☐	☒	The exact sample size (n) for each experimental group/condition, given as a discrete number and unit of measurement
☐	☒	A statement on whether measurements were taken from distinct samples or whether the same sample was measured repeatedly
☒	☐	The statistical test(s) used AND whether they are one- or two-sided<br>*Only common tests should be described solely by name; describe more complex techniques in the Methods section.*
☒	☐	A description of all covariates tested
☒	☐	A description of any assumptions or corrections, such as tests of normality and adjustment for multiple comparisons
☒	☐	A full description of the statistical parameters including central tendency (e.g. means) or other basic estimates (e.g. regression coefficient) AND variation (e.g. standard deviation) or associated estimates of uncertainty (e.g. confidence intervals)
☒	☐	For null hypothesis testing, the test statistic (e.g. F, t, r) with confidence intervals, effect sizes, degrees of freedom and P value noted<br>*Give P values as exact values whenever suitable.*
☒	☐	For Bayesian analysis, information on the choice of priors and Markov chain Monte Carlo settings
☒	☐	For hierarchical and complex designs, identification of the appropriate level for tests and full reporting of outcomes
☒	☐	Estimates of effect sizes (e.g. Cohen's d, Pearson's r), indicating how they were calculated

Our web collection on statistics for biologists contains articles on many of the points above.

Software and code

Policy information about availability of computer code

Data collection	All finite element simulations were performed using COMSOL Multiphysics commercial software (version 5.3a, COMSOL Inc., Burlington, MA). User-defined equations are described in Materials and Methods, and model files have been made available in a public repository, as described in the Data Availability section.
Data analysis	Bruker Paravision 5.1 software was used to calculate diffusion tensors from diffusion-weighted MRI data. MRI analysis utilized the previously published software packages ITK-SNAP (http://www.itksnap.org) and CARET (http://brainvis.wustl.edu), as described in Materials and Methods. Custom codes created in MATLAB (version R2020a) were used for further analysis and visualization. These codes have been made available in a public repository, as described in the Data Availability section.

For manuscripts utilizing custom algorithms or software that are central to the research but not yet described in published literature, software must be made available to editors and reviewers. We strongly encourage code deposition in a community repository (e.g. GitHub). See the Nature Research guidelines for submitting code & software for further information.

Data

Policy information about availability of data

All manuscripts must include a data availability statement. This statement should provide the following information, where applicable:

- Accession codes, unique identifiers, or web links for publicly available datasets
- A list of figures that have associated raw data
- A description of any restrictions on data availability

The data that support these findings, including model files and processed MRI data generated in this study, have been deposited in the Zenodo database, DOI: 10.5281/zenodo.5573193. Source data for plots in Fig. 7c-d are provided with this paper.

Field-specific reporting

Please select the one below that is the best fit for your research. If you are not sure, read the appropriate sections before making your selection.

☒ Life sciences ☐ Behavioural & social sciences ☐ Ecological, evolutionary & environmental sciences

For a reference copy of the document with all sections, see nature.com/documents/nr-reporting-summary-flat.pdf

Life sciences study design

All studies must disclose on these points even when the disclosure is negative.

Sample size	This study focused on detailed analysis of unique, high resolution DTI data in the developing rhesus macaque, manually reconstructed and analyzed using previously described methods. Detailed analysis of individual subjects was chosen to illustrate the striking consistency was observed across the brain for these examples. A small sample was sufficient for this illustration and no sample size calculation was necessary. Sample sizes in this study were chosen based on inclusion of all available data to date.
Data exclusions	No data were excluded from the analysis.
Replication	For the major trend of interest, analysis was repeated in three subjects at a similar stage of development, confirming the reproducibility of experimental findings, as illustrated in Fig. 5.
Randomization	Since this study did not consider an experimental group, randomization was not relevant to this study.
Blinding	Since this study did not consider an experimental group, blinding was not relevant to this study.

Reporting for specific materials, systems and methods

We require information from authors about some types of materials, experimental systems and methods used in many studies. Here, indicate whether each material, system or method listed is relevant to your study. If you are not sure if a list item applies to your research, read the appropriate section before selecting a response.

Materials & experimental systems

n/a	Involved in the study	
☒	☐	Antibodies
☒	☐	Eukaryotic cell lines
☒	☐	Palaeontology and archaeology
☐	☒	Animals and other organisms
☒	☐	Human research participants
☒	☐	Clinical data
☒	☐	Dual use research of concern

Methods

n/a	Involved in the study	
☒	☐	ChIP-seq
☒	☐	Flow cytometry
☐	☒	MRI-based neuroimaging

Animals and other organisms

Policy information about studies involving animals; ARRIVE guidelines recommended for reporting animal research

Laboratory animals	Ex vivo MRI scans were performed on four rhesus monkey brains, one from a control fetus perfusion fixed at gestational week 85 (female), and three from three other control fetuses perfusion fixed at gestational week 110 (2 female, 1 male).
Wild animals	No wild animals were used in the study.
Field-collected samples	No field collected samples were used in the study.
Ethics oversight	All procedures for generating tissue used in this study were conducted in accordance with the Guide for the Care and Use of Laboratory Animals and the National Institutes of Health Guidelines for the Care and Use of Laboratory Animal resources and approved by the Oregon National Primate Research Center (ONPRC) Institutional Animal Care and Use Committee.

Note that full information on the approval of the study protocol must also be provided in the manuscript.

Magnetic resonance imaging

Experimental design

Design type	This is a study of normal development.

Design specifications | 1 diffusion MRI dataset per tissue sample.

Behavioral performance measures | N/A

Acquisition

Imaging type(s) | Diffusion

Field strength | 12

Sequence & imaging parameters | Multi-slice spin echo Stejskal-Tanner pulse sequence.

Area of acquisition | Whole hemisphere, post mortem.

Diffusion MRI ☒ Used ☐ Not used

Parameters | 3 b=0, 25 b=2.5 ms/micrometer^2 images. All images were acquired in the same phase of the cardiac cycle.

Preprocessing

Preprocessing software | Bruker Paravision 5.1 software was used to calculate diffusion tensors from diffusion-weighted MRI data.

Normalization | Images were analyzed in the "native" space.

Normalization template | N/A

Noise and artifact removal | N/A

Volume censoring | N/A

Statistical modeling & inference

Model type and settings | N/A

Effect(s) tested | N/A

Specify type of analysis: ☒ Whole brain ☐ ROI-based ☐ Both

Statistic type for inference (See Eklund et al. 2016) | N/A

Correction | N/A

Models & analysis

n/a	Involved in the study
☒	☐ Functional and/or effective connectivity
☒	☐ Graph analysis
☒	☐ Multivariate modeling or predictive analysis
